# Supplementary material for: Characterisation of a synthetic Archeal membrane reveals a possible new adaptation route to extreme conditions
Source: Commun Biol. 2021 Jun 2;4:653. doi: 10.1038/s42003-021-02178-y (PMC8172549; doi:10.1038/s42003-021-02178-y)
Supplement: Supplementary file 2 — Supplementary Information [file 42003_2021_2178_MOESM2_ESM.pdf]

Supplementary figures

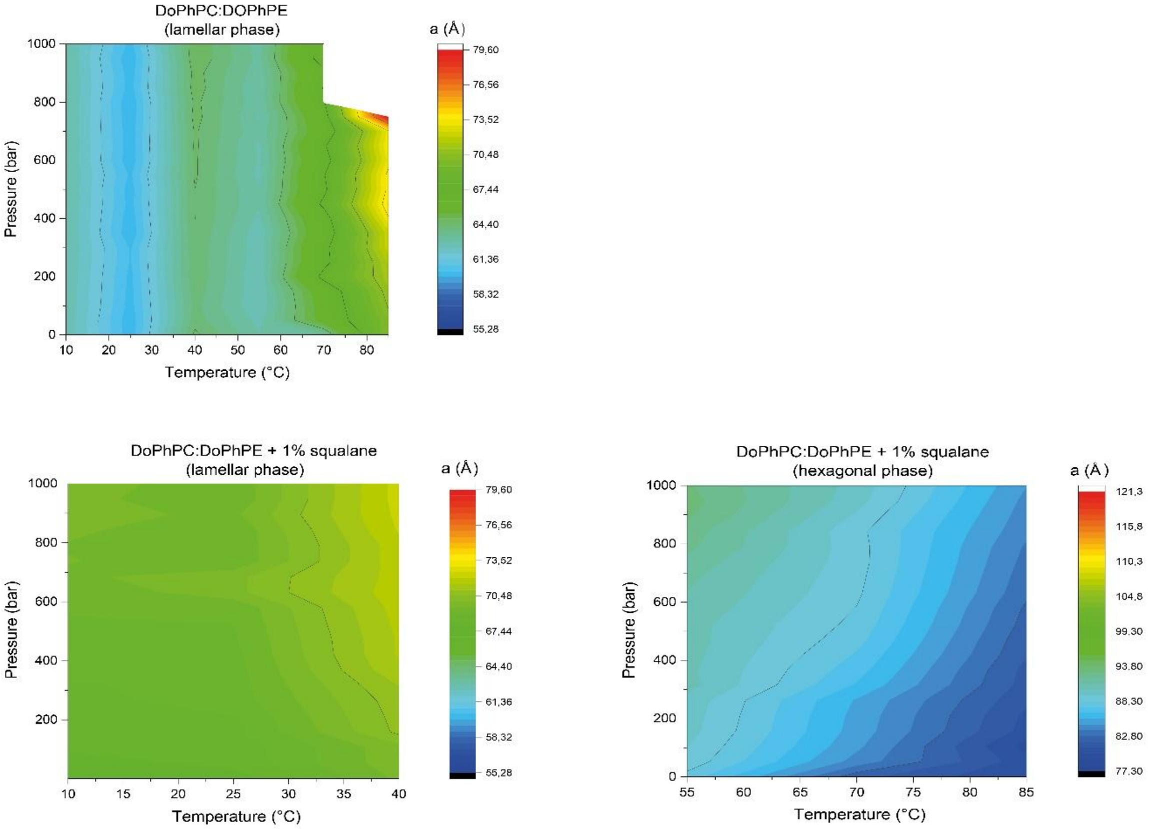

Supplementary Figure S1. Lattice parameters of lipid phases are modulated by temperature and pressure. Two-dimensional pressure-temperature colour maps of lattice parameters (d-spacing) for lamellar phases of DohPC:DoPhPE (9:1) (top left) and with 1 mol% squalane (bottom left) and for hexagonal phases of DoPhPC:DoPhPE (9:1) + 1 mol% squalane (right). In the absence of squalane, the membrane is highly unstructured at extreme conditions (70°C – 85°C), and it was not possible to determine its d-spacing in the most extreme conditions (85°C and 700 bar). However, in the presence of squalane, the membrane is highly structured under extreme conditions and presents non-lamellar phases, such as the hexagonal phase.

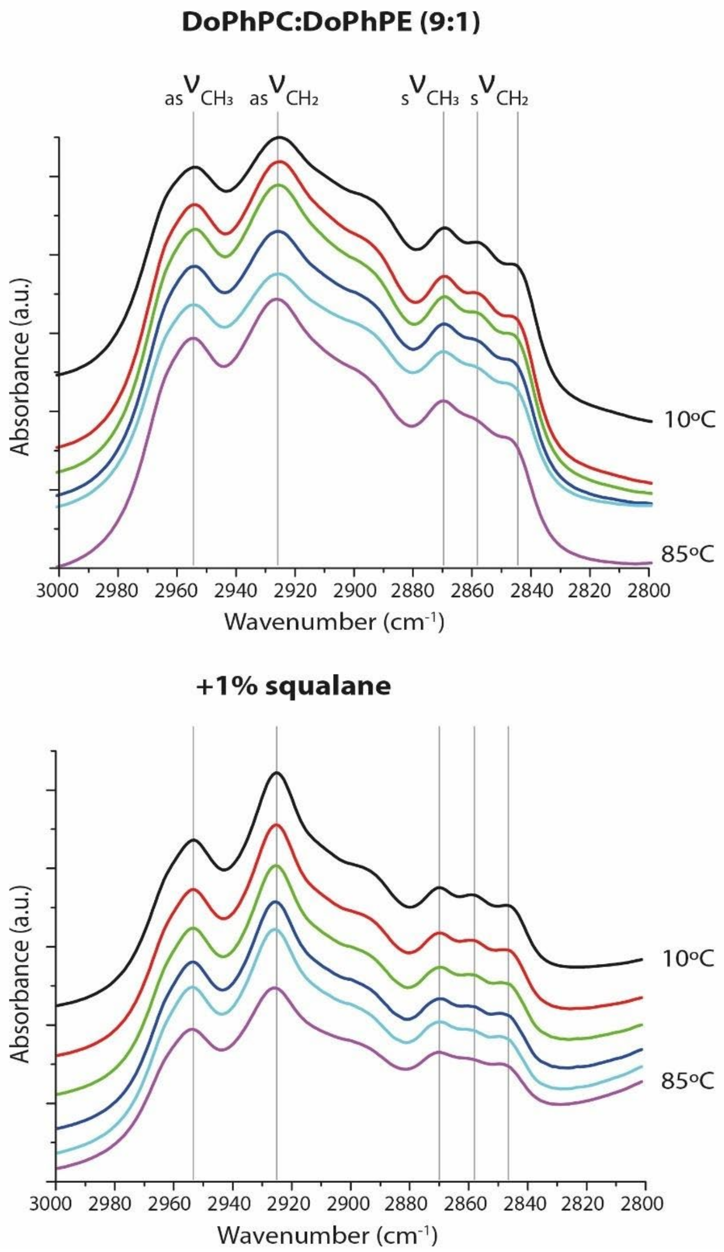

Supplementary FigureS2. Intramolecular and intermolecular vibrations are slightly sensitive to temperature and the presence of squalane. Infrared spectra of DoPhPC:DoPhPE(9:1) (above) and DoPhPC:DoPhPE(9:1) with 1 mol% squalane (below) at ambient pressure and different temperatures (black: 10ºC, red: 25ºC, green: 40ºC, blue: 55ºC, cyan: 70ºC, magenta: 85ºC). Asymmetric CH_3 stretching corresponds to approximately 2955 cm-1, asymmetric CH_2 stretching to approximately 2925 cm^{-1}, symmetric CH_2 stretching to approximately 2870 cm^{-1}, and symmetric CH_2 stretching to the double peak at approximately 2845 cm^{-1} and 2855 cm^{-1}.

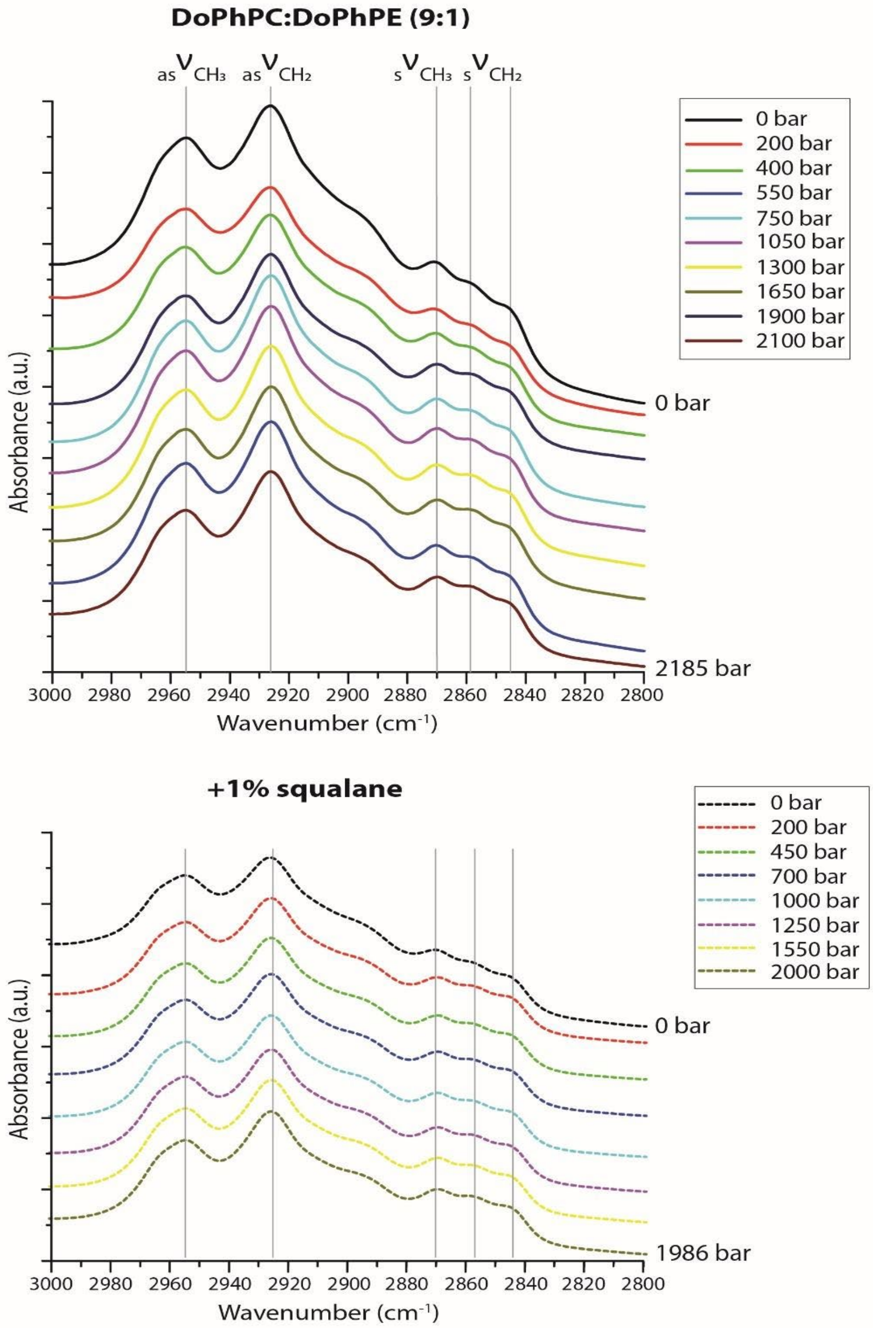

Supplementary Figure S3. Lipid molecular vibrations are slightly sensitive to pressure and the presence of squalane. Infrared spectra of DoPhPC:DoPhPE (9:1) (above) and DoPhPC:DoPhPE (9:1) with 1 mol% squalane (below) at 25°C and different applied pressures (black solid line: 0 bar, red solid line: 200 bar, green solid line: 400 bar, blue solid line: 550 bar, cyan solid line: 750 bar, magenta solid line: 1000 bar, yellow solid line: 1330 bar, ochre solid line: 1650 bar, dark blue solid line: 1900 bar, brown solid line: 2100 bar, black dashed line: 0 bar, red dashed line: 200 bar, green dashed line: 450 bar, blue dashed line: 700 bar, cyan dashed line: 1000 bar, magenta dashed line: 1250 bar, yellow dashed line: 1550 bar, ochre dashed line: 2000 bar). Asymmetric CH_3 stretching corresponds to approximately 2955 cm^{-1}, asymmetric CH_2 stretching to approximately 2925 cm^{-1}, symmetric CH_2 stretching to approximately 2870 cm^{-1} and symmetric CH_2 stretching to the double peak at approximately 2845 cm^{-1} and 2855 cm^{-1}.

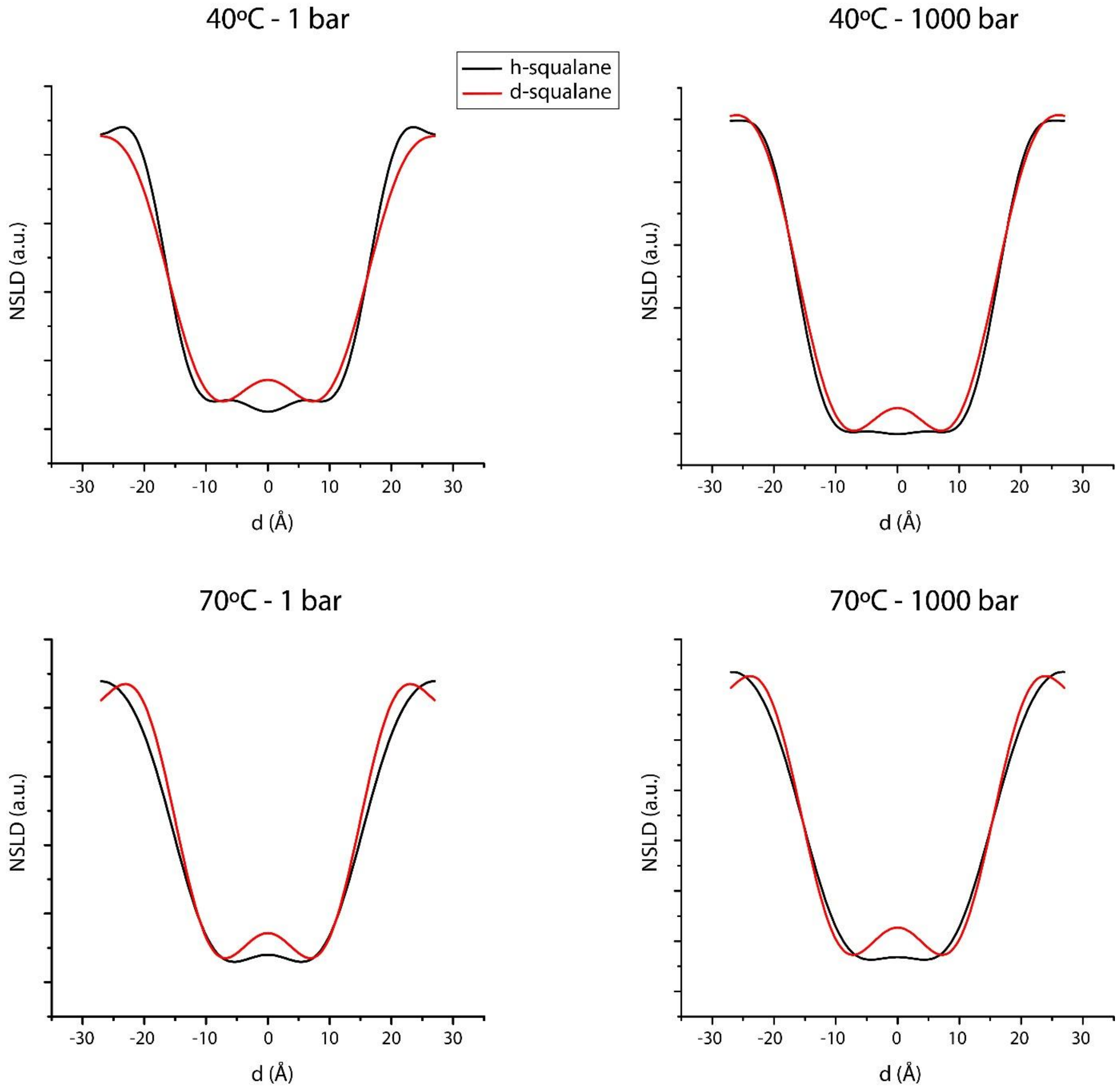

Supplementary Figure S4. Squalane is placed in the midplane of the bilayer, perpendicular to the lipids, at high temperatures and high hydrostatic pressures. Neutron scattering length densities of DoPhPC:DoPhPE (9:1) with 1 mol % hydrogenated squalane (black) and 1 mol% deuterated squalane (red) at 40°C, 70° C and 1 bar and 1000 bar. These NSLDs confirm that squalane is positioned at the midplane of the bilayer, even at high temperature and high pressure.

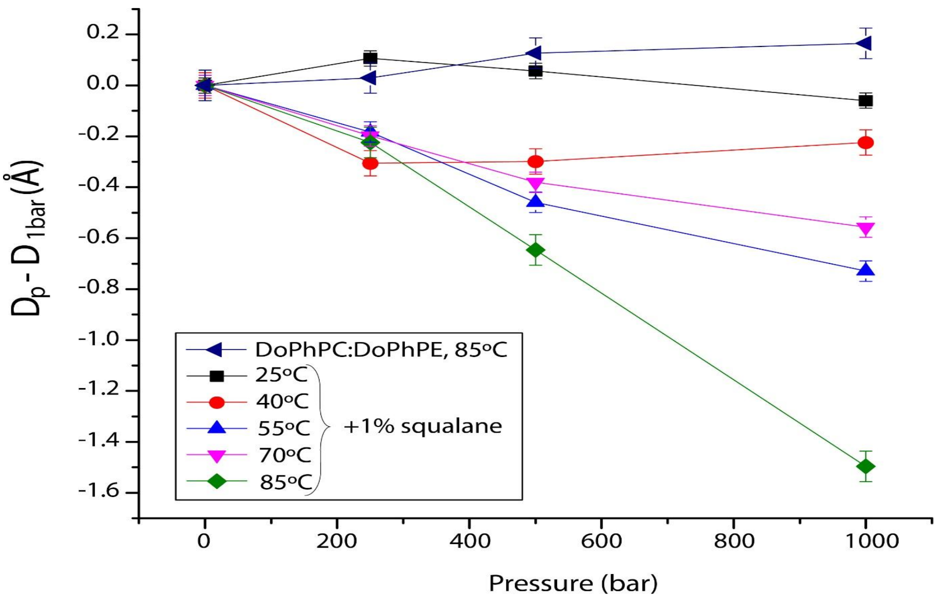

Supplementary Figure S5. In the presence of squalane, especially at high temperatures, the lipid bilayer is highly compressible. Variation in the lamellar lattice parameters (Dp: d-spacing at different pressures (1 bar, 250 bar, 500 bar, 1000 bar), D1bar: d-spacing at 1 bar) obtained by multistacking bilayers from neutron diffraction, measured at different pressures for DoPhPC:DoPhPE at 85°C (blue leftward-pointing triangles) and for DoPhPC:DoPhPE (9:1) +1 mol% squalane at 25°C (black squares), 40°C (red spheres), 55°C (blue upward-pointing triangles), 70°C (magenta downward-pointing triangles), and 85°C (green diamonds).

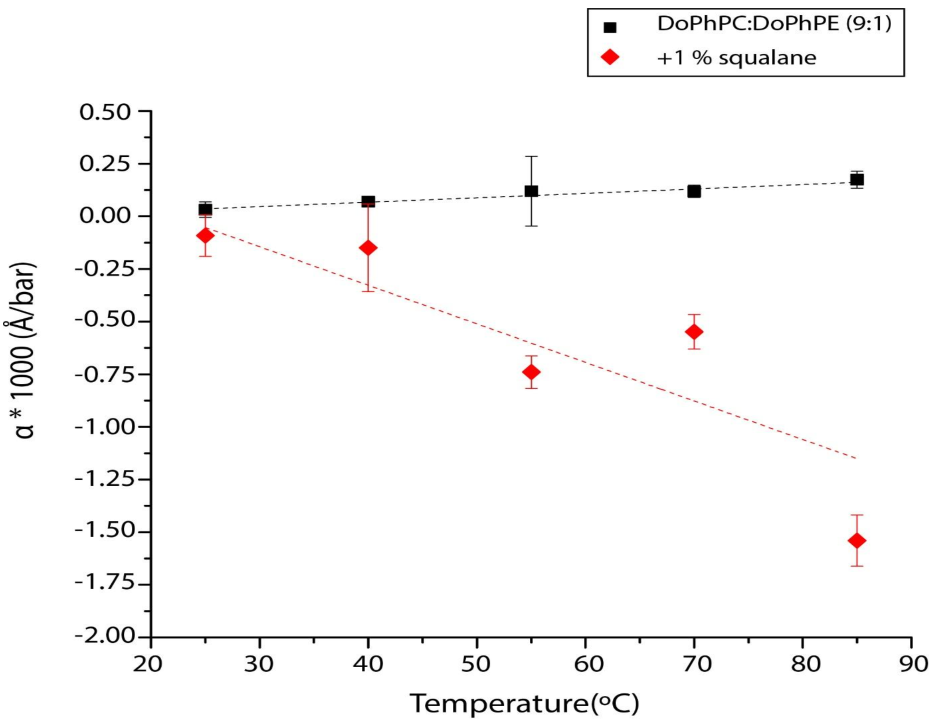

Supplementary Figure S6. The compressibility of the lipid bilayer in the presence of squalane tends to increase in proportion to temperature. The task is representation of the slope (α) of the pressure dependence of the d-spacing for different temperatures (Figure S6). The value is multiplied by 1000 to facilitate better visualization. Data are shown for DoPhPC:DoPhPE (9:1) in the absence (black squares) and presence 1 mol% squalane (red triangles). In contrast to the neat lipid mixture, in the presence of squalane, pressure decreases the bilayer thickness, and this effect is more pronounced at high temperatures. Dashed lines are linear fits to guide the eyes.

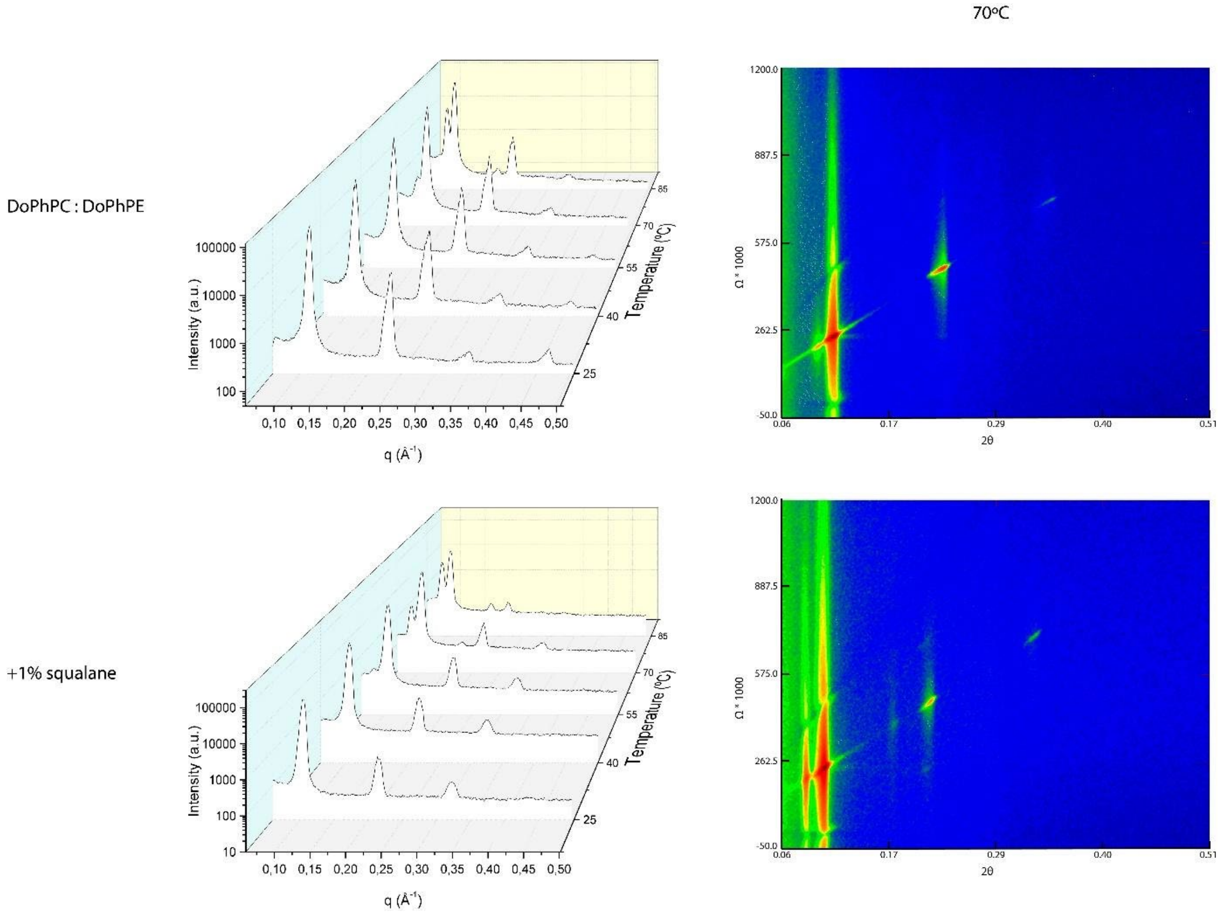

Supplementary Figure S7. Squalane facilitates the formation of new lipid phases. Left side, intensities obtained by the neutron diffraction of DoPhPC:DoPhPE (9:1) in the absence and presence of 1 mol% squalane at different temperatures (25° C, 40°C, 55°C, 70°C and 85°C). The corresponding 2D diffraction patterns at 70°C show the coexistence of two lamellar phases in different proportions, which confirms that squalane promotes the emergence of new phases while increasing membrane stability under extreme conditions (*e.g.*, 70°). The presence of this new phase may be correlated with the laurdan data which shows the slow replacement between to lamellar phases. The fact that it is also visible at high temperature by neutron diffraction indicates that it is sufficiently ordered spatially to be detectable. It also shows that it is stable at higher temperatures than the phase present at low temperature. Hence, it has real significance in terms of adaptation to high temperature. Furthermore, the coexistence of stable phases may help the differentiation of the membrane in to functional domains, which is important in terms of adaptation to stress, and a well-known feature of eukaryote and bacterial cell membranes, which needs to be demonstrated in Archaea.
